# Vestibular-dependent inter-stimulus interval effects on sound evoked potentials of central origin

**DOI:** 10.1016/j.heares.2016.07.017

**Published:** 2016-11

**Authors:** N.P.M. Todd, S. Govender, J.G. Colebatch

**Affiliations:** a Department of Psychology, University of Exeter, Exeter EX4 4QG, UK; b Prince of Wales Clinical School and Neuroscience Research Australia, University of New South Wales, Randwick, Sydney, NSW 2052, Australia

**Keywords:** Auditory evoked potentials (AEPs), Vestibular evoked myogenic potentials (VEMPs), Vestibular evoked potentials (VsEPs), Locomotion

## Abstract

Todd et al. (2014ab) have recently demonstrated the presence of vestibular-dependent contributions to auditory evoked potentials (AEPs) when passing through the vestibular threshold as determined by vestibular evoked myogenic potentials (VEMPs), including a particular deflection labeled as an N42/P52 prior to the long-latency AEPs N1 and P2. In this paper we report the results of an experiment to determine the effect of inter-stimulus interval (ISI) and regularity on potentials recorded above and below VEMP threshold. Five healthy, right-handed subjects were recruited and evoked potentials were recorded to binaurally presented sound stimulation, above and below vestibular threshold, at seven stimulus rates with ISIs of 212, 300, 424, 600, 848, 1200 and 1696 ms. The inner five intervals, i.e. 300, 424, 600, 848, 1200 ms, were presented twice in both regular and irregular conditions. ANOVA on the global field power (GFP) were conducted for each of four waves, N42, P52, N1 and P2 with factors of intensity, ISI and regularity. Both N42 and P52 waves showed significant ANOVA effects of intensity but no other main effects or interactions. In contrast both N1 and P2 showed additional effects of ISI, as well as intensity, and evidence of non-linear interactions between ISI and intensity. A source analysis was carried out consistent with prior work suggesting that when above vestibular threshold, in addition to bilateral superior temporal cortex, ocular, cerebellar and cingulate sources are recruited. Further statistical analysis of the source currents indicated that the origin of the interactions with intensity may be the ISI sensitivity of the vestibular-dependent sources. This in turn may reflect a specific vestibular preference for stimulus rates associated with locomotion, i.e. rates close to 2 Hz, or ISIs close to 500 ms, where saccular afferents show increased gain and the corresponding reflexes are most sensitive.

## Highlights

• Effect of inter-stimulus interval on sound evoked potentials measured at intensities above and below vestibular threshold.
• Evidence was found for vestibular-dependent non-linear intensity by inter-stimulus interval interactions.
• The non-linear effects may reflect vestibular sensitivity for stimulus rates of around 2 Hz associated with locomotion.

## 1 Introduction

Acoustic sensitivity of the human vestibular system has long been established and can be demonstrated by means of evoked electromyographic (EMG) signals (Bickford et al., 1964). Such EMG responses can be measured either from muscles of the neck, e.g. the sternocleidomastoid muscle, reflecting the vestibular-collic reflex pathways (the vestibular evoked myogenic potential or VEMP (Colebatch et al., 1994)) or from extra-ocular eye muscles, reflecting activation of the vestibular ocular reflex pathways (ocular VEMP or OVEMP (Rosengren et al., 2005, Todd et al., 2007)).

In the last decade evidence has accumulated from electroencephalographic (EEG) studies in humans that vestibular receptors may also contribute to sound evoked potentials of central origin. Following a study by de Waele et al. (2001), who showed the existence of short-latency potentials (8–15 ms) in response to electrical stimulation, Todd et al. (2003) demonstrated a similar response to 500 Hz bone-conducted (BC) sound. These acoustically evoked short-latency vestibular evoked potentials (VsEPs) were confirmed to have vestibular origin as they were absent in avestibular patients but present in deaf subjects with intact VEMPs (Rosengren and Colebatch, 2006). Later Todd et al. (2008) used source analysis to confirm that the short-latency VsEPs produced by air-conducted (AC) and BC sound are dominated by the pathways underlying the vestibular-ocular reflex, but also suggested activity in frontal cortex. More recently McNerney et al. (2011) used an alternative source analysis method to suggest that a wider range of vestibular cortical areas contribute to the short-latency potentials activated by sound.

Most recently Todd et al. (2014a) provided evidence that in addition to short-latency effects there were likely vestibular contributions at longer latencies for AC sound stimulation. These were recognized by systematic changes that took place in morphology and by the intensity dependence of the responses in passing through the vestibular threshold. Of particular interest was a medium-latency deflection, labelled N42/P52, which exhibited a change in slope and latency function, and was absent in an avestibular patient. The long-latency auditory evoked potential (LAEP) N1 also showed some changes in passing through the VEMP threshold. A source analysis indicated a possible contribution of cingulate cortex to both the N42 and N1, as well as temporal lobe, cerebellar and other sub-cortical sources. A follow-up study comparing left vs right stimulation showed that the vestibular-dependent responses indicated a left-ear/right-hemisphere advantage for the long-latency responses (Todd et al., 2014b). Source analysis indicated that these effects may mediated by a contralateral projection to the right cingulate cortex. In addition we found evidence of a possible vestibular contribution to the auditory T-complex in radial temporal lobe sources.

It has been well-established that the LAEPs and their magnetic equivalents are highly dependent on the inter-stimulus interval (ISI) (Picton et al., 1974, Hari et al., 1982, Lü et al., 1992, Sams et al., 1993, Carver et al., 2002; Snyder and Large, 2005). In the above studies by Todd et al., 2014a, Todd et al., 2014b, however, the ISIs were limited to a narrow range between 600 and 1000 ms but any possible vestibular interactions with the ISI dependency are unknown. We wished in the present study, therefore, to investigate the effects of ISI and regularity on the vestibular-dependent changes in sound evoked potentials, and in particular the N42/P52, N1 and P2 waves. The presence of a vestibular interaction with ISI would be of considerable theoretical interest, for example with respect to suggested vestibular contributions to rhythm perception (Trainor et al., 2009, Todd and Lee, 2015a, Todd and Lee, 2015b). Knowledge of such an interaction may also have benefit for clinical usage of VsEPs and for determining optimal stimulus rates.

## 2 Material and methods

### 2.1 Subjects

Five healthy subjects were selected for this study (2 females and 3 males, age range 22–53). All subjects were first screened for any hearing impairment using a standard screening audiometer (frequency range 250 Hz to 8 kHz) in order to check that their hearing thresholds were in the normal range. Prior to any testing, all participants gave written informed consent according to the Declaration of Helsinki.

### 2.2 Stimuli

The experimental stimuli employed for obtaining vestibular responses were AC 2-ms, 500-Hz, single cycle tone pips. AC stimuli were delivered by insert earphones (3A insert earphone, E-A-RTone Gold, Guymark UK Limited). Stimulus calibration was carried out using a GRAS IEC711 Coupler (RA0045) and a pressure-field microphone (Model 4134) with a 2260 Investigator (Brüel and Kjaer, Naerum, Denmark). The stimuli were generated using customised software with a laboratory interface (power 1401, Cambridge Electronic Design, Cambridge, UK) and a commercial or custom amplifier. Two intensities were employed, i.e. +6–9 dB re 1 V peak (133–136 dB pk SPL) and −30 dB re 1 V pk, arranged so that the high intensity stimulus was above vestibular threshold and the low intensity stimulus below threshold, as is commonly assumed in vestibular research (e.g. McNerney et al., 2011).

### 2.3 Vestibular responses

As only two intensities were employed in the present study we did not measure exact vestibular thresholds, but checked prior to conducting EEG that VEMPs were present and absent for the two intensities. Subjects were tested lying supine on a couch, with the backrest tilted to approximately 30–45° from the horizontal, and required to lift their heads against gravity to activate the sternocleidomastoid (SCM) muscles. Surface EMG was measured from the ipsilateral SCM using self-adhesive Ag/AgCl electrodes. Active surface electrodes were placed over the middle of the SCM muscle belly and were referred to electrodes placed on the medial clavicle. EMG was amplified, bandpass filtered (5 Hz–1 kHz) and sampled using a Power1401 interface (CED Ltd., Cambridge, UK). The EMG was sampled at a rate of 5 kHz, starting 10 ms before to 80 ms following stimulus onset, and averaged. Up to 200 stimuli were presented at a stimulus rate of about 5 Hz. The presence or absence of a VEMP was determined by visual inspection.

### 2.4 VsEPs

VsEPs were recorded with subjects comfortably seated with their gaze directed straight ahead to picture card at a viewing distance of 100 cm. AC pips were presented binaurally at seven stimulus rates with ISIs of 212, 300, 424, 600, 848, 1200 and 1696 ms. The inner five intervals, i.e. 300, 424, 600, 848, 1200 ms, were presented twice in both regular and irregular conditions. In the irregular conditions stimuli had ISIs randomly varying with a uniform probability distribution between adjacent intervals, thus for the 300 ms irregular condition the ISIs varied randomly between 212 and 424 ms. Evoked potentials (EPs) were recorded for the two intensities. EEG was recorded using a 64-channel EEG system (Biosemi, Inc., USA). Additional electrodes were also placed below each eye (i.e. infra-ocular electrodes, IO1 and IO2), at deep frontal (F9 and F10) and at ear-lobe locations (A1 and A2). Electrode offset (i.e. running average of the voltage measured between CMS and each active electrode) was maintained below 20 μV. Recordings were made with a band-pass of between 0.16 Hz and 1 kHz. Artefact elimination, epoching and averaging of EPs were carried out using the BESA 5 software. Epochs were 350 ms in length, from 50 ms before to 300 ms following the stimulus onset. After collection, EPs were filtered at 1–300 Hz and referenced either to linked ear-lobe electrodes or to an average reference using Scan software (v4.3, Neuroscan, USA). All subsequent analyses were conducted using the average reference.

### 2.5 Source analyses

BESA software (version 5.3 MEGIS Software GmbH, Germany) was used for dipole modelling. The standard four-shell elliptical head approximation was employed with the following parameters. The radial thickness of the head, scalp, bone and CSF were 85, 6, 7 and 1 mm, respectively, with conductivities set to 0.33, 0.33, 0.0042 and 1.0, respectively. We adopted a modelling strategy from previous occasions of using pairs of regional sources and dipoles (Todd et al., 2008, Todd et al., 2014a, Todd et al., 2014b). This approach had been arrived at after extensive modelling using different strategies. Ocular sources and temporal lobe sources are ubiquitous for the stimuli employed and two pairs locate without fail to these areas, irrespective of starting conditions. Regional sources are appropriate to model the complexity of the (known) activation of the bilateral extra-ocular eye muscles (EOM) in conjunction with the retinal corneal dipole (RCD) associated with eye movement, and for activity in bilateral temporal cortex, which includes independent radial and tangential components (Scherg et al., 1989, Näätänen and Picton, 1987). For the additional dipole pair sources no constraint was applied other than symmetry, the starting point for these being determined by previous solutions indicating anterior and posterior regions, with the ocular and temporal sources starting from their original positions from the lower order solutions. The bilateral constraint is only an approximation to bilateral auditory activation but has the advantage of reducing the number of possible solutions and is a standard approach modelling AEPs (Scherg and Von Cramon, 1986, Näätänen and Picton, 1987, Scherg et al., 1989).

### 2.6 Statistical analyses

Repeated measures ANOVA were carried out on measurements of RMS GFP at the response peaks corresponding to the Nb/N42, P52/P1, N1 and P2 (SPSS ver. 22, IBM Corp.). These were conducted separately for each wave for the regular conditions over the seven interval range with ISI and intensity as within-subjects factors, and then again for the five interval range with ISI, intensity and regularity as within-subjects factors. We also conducted a separate series of ANOVA on the source currents after a suitable BESA model was obtained. In previous studies we have carried out a log-transform on the dependent measures as it is generally the case that VEMPs and VsEPs obey a power law as a function of intensity (Dennis et al., 2014). In the present study all analyses were conducted using non-transformed measurements because for the source currents the log transform produced a risk of outliers from some small current values.

## 3 Results

### 3.1 Properties of the averaged electroencephalography (EEG)

**Fig. 1:**
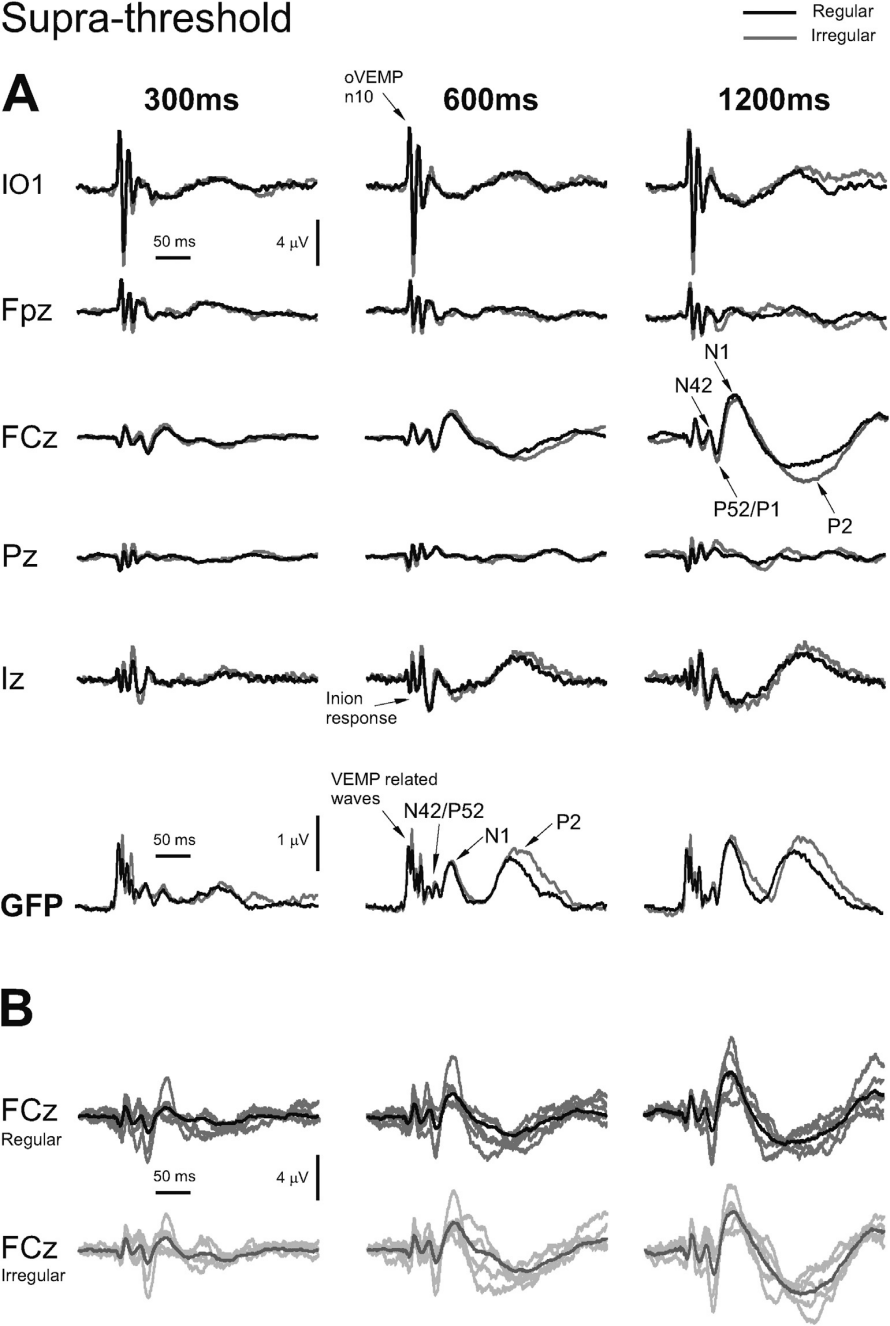
(A) Grand means of evoked potentials produced by binaural stimulation with 500 Hz, 2 ms pips at +6–9 dB re 1 V with inter-stimulus of 300, 600 and 1200 ms from selected electrodes IO1, Fpz, FCz, Pz and Iz in 5 healthy subjects. For each electrode location the two traces show the regular (black) vs. irregular conditions as black and grey traces respectively. All electrodes are referred to an average reference and the global field power (GFP) is also indicated. (B) Individual subject evoked potentials measured at FCz compared with the grand mean for regular (dark grey) vs. irregular (light grey) conditions.

**Fig. 2:**
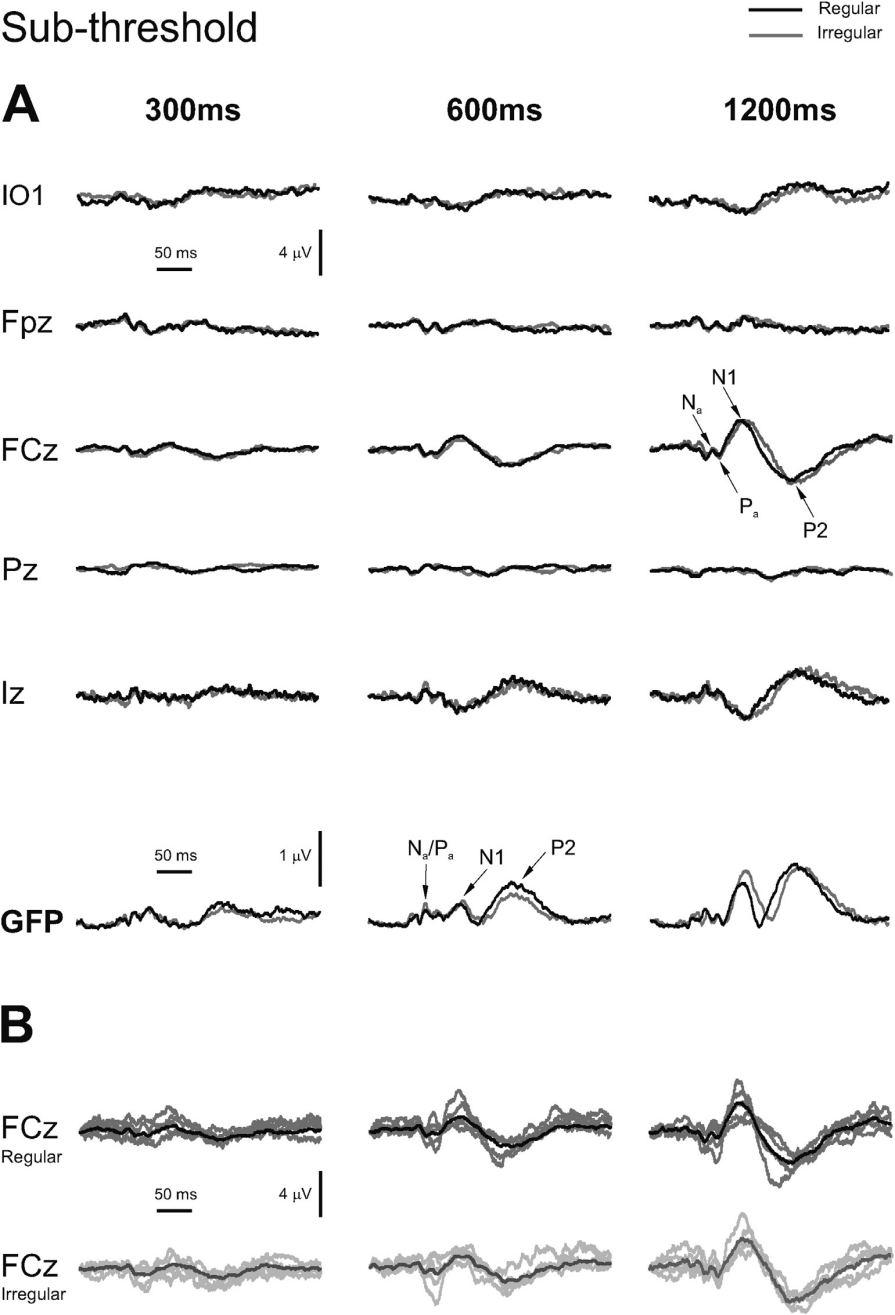
(A) Grand means of evoked potentials produced by binaural stimulation with 500 Hz, 2 ms pips at −30 dB re 1 V with inter-stimulus of 300, 600 and 1200 ms from selected electrodes IO1, Fpz, FCz, Pz and Iz in 5 healthy subjects. For each electrode location the two traces show the regular (black) vs. irregular conditions as black and grey traces respectively. All electrodes are referred to an average reference and the global field power (GFP) is also indicated. (B) Individual subject evoked potentials measured at FCz compared with the grand mean for regular (dark grey) vs. irregular (light grey) conditions.

Grand means for EPs produced by regular vs irregular presentations for illustrative ISIs of 300, 600 and 1200 ms in selected electrodes at supra- vs sub-threshold intensities are shown in Fig. 1A (supra) and Fig. 2A (sub) respectively. The grand means are representative of the individual responses, as illustrated in Fig. 1, Fig. 2B. As previously reported (Todd et al., 2014a, Todd et al., 2014b), the sub-threshold conditions show a typical AEP pattern consisting of mid-latency (MLR) Na and Pa waves followed by the long latency (LAEP) N1 and P2 waves, well illustrated in channel FCz (Fig. 2A). In contrast, the supra-threshold condition shows the expected changes in morphology. These are characterised by short-latency waves, which have no auditory correlate, the OVEMP and inion related responses N10 and P10 (Fig. 1), and a later deflection, labelled N42/P52 followed by the LAEP N1 and P2. The supra- vs. sub-threshold waves also show a small latency shift.

### 3.2 Source analyses

**Fig. 3:**
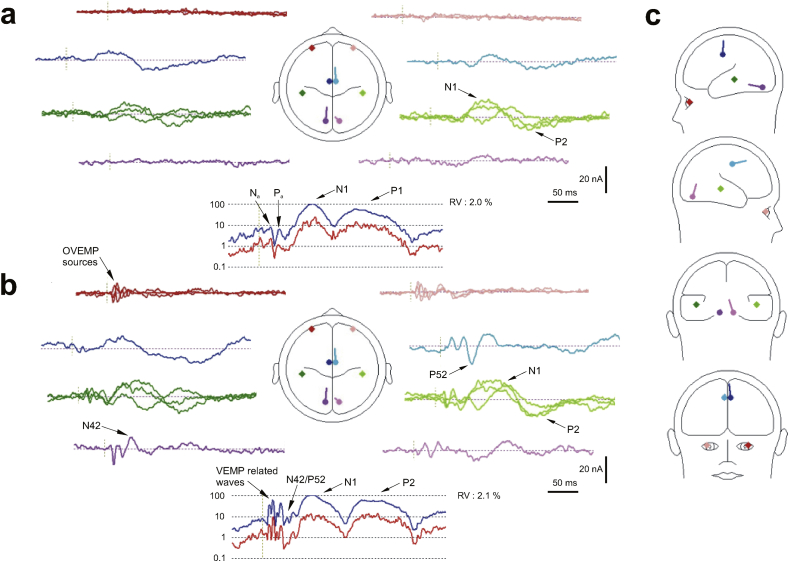
(a) An illustration of the BESA model (Table 1) for supra-threshold intensity stimulation at the regular 1696 ms ISI condition (b) the same model response for the sub-threshold intensity stimulation at the regular 1696 ms ISI condition. (c)Sagittal and coronal views of the eight source locations. Occular sources in hues of red, cingulate source in hues of blue, temporal lobe sources in hues of green and cerebellar sources in hues of mauve (see Table 1).

Application of our standard approach to the longest latency condition with ISI of 1696 ms, i.e. the condition with the largest magnitude responses, yielded solutions very similar to those found previously (Table 1). These consisted of a pair of regional sources located in the superior temporal gyrus, a pair of regional sources located in the bilateral orbits, a pair of dipoles located within the cingulate gyrus and a pair located within the cerebellum. As before the analysis was carried out over the whole epoch, in this case from 7 to 300 ms and with a band-pass of 1–300 Hz. Fig. 3 illustrates the model as applied to the ISI 1696 ms condition at supra and sub-threshold intensities with the source TTCs given in Table 1. Consistent with previous observations for the supra-threshold condition the ocular and cerebellar (i.e. sub-cortical) sources show early bilateral activation corresponding with the short-latency VsEPs while the cortical sources show greater activity for the later components of the evoked response. In contrast for the sub-threshold condition there is little or no early activity, especially in the sub-cortical sources. The cortical sources continue to show significant activity during the late AEP P1, N1 and P2 waves.

### 3.3 Statistical analyses of changes in the GFP

Fig. 1, Fig. 2 above also illustrate the global field power (GFP) for each of the conditions and points where measurements were made for individual subjects. Although there was some latency shift with the intensity condition we used the peak in the mean GFP for each condition to determine the measurement point for the individual subjects, i.e. the peak in the individual GFP waveform closest to the mean GFP. We report here first effects on the GFP using μV for the short-latency waves Nb/N42 and Pb/P52. For regular ISIs two within-subjects factors were employed, i.e. intensity (2 levels) and ISI (7 levels), and for irregular ISIs an additional third factor of regularity (2 levels).

**Fig. 4:**
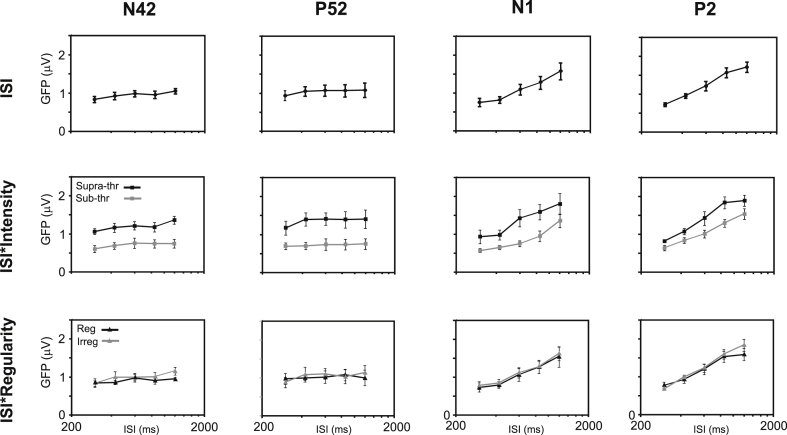
Estimated marginal means (with standard errors (SEs)) from ANOVAs of GFP for five irregular inter-stimulus intervals (ISIs) measured at latencies of the N42, the P52, the N1 and the P2. (Top) Main effect of ISI, (middle) effect of intensity by ISI and (bottom) effect of regularity by ISI. ISIs are presented on a log-scale from 200 to 2000 ms.

For the Nb/N42 although there appeared to be a small trend to increase in magnitude with ISI and some evidence of a preference for 600 ms (Fig. 4), there were no significant main ANOVA effects of ISI or regularity (see also Table 2). There was though a main effect of intensity F_(1,4)_ = 15.2, *p* < 0.05 and F_(1,4)_ = 23.1, *p* < 0.01 respectively for the seven regular and five irregular ISI conditions. No significant interactions were obtained. Similarly the ANOVA carried out for the Pb/P52 wave showed no main effects of ISI nor of regularity, but again a significant main effect of intensity, F_(1,4)_ = 34, *p* < 0.05 and F_(1,4)_ = 36, *p* < 0.005 respectively for the seven regular and five irregular ISI conditions (Fig. 4, Table 2). Thus both N42 and P52 waves showed main effects of intensity with no other significant effects or interactions. The change of stimulus intensity produced about a doubling in magnitude of the GFP.

Turning now to the long latency waves, for the N1 an ANOVA on the GFP for the seven regular ISI conditions yielded main effects of both ISI (F_(6,24)_ = 23, *p* < 0.001), and intensity (F_(1,4)_ = 24, *p* < 0.05). When an ANOVA was applied to the five cases for which N1 responses were also recorded to irregular ISIs, main effects were observed again for both ISI (F_(4,16)_ = 17.5, *p* < 0.005), and intensity (F_(1,4)_ = 16.7, *p* < 0.05). There was though no significant main effect of regularity, nor were there any significant interactions. However, although the ISI by intensity interaction did not reach significance, there were significant quadratic (F_(1,4)_ = 19.3, *p* < 0.05) and cubic (F_(1,4)_ = 50.5, *p* < 0.005) ISI by intensity contrasts. These effects are illustrated in Fig. 4 and Table 2. The P2 showed a broadly similar pattern of significance in the main effects of intensity and ISI, respectively F_(1,4)_ = 37.1, *p* < 0.005 and F_(1,4)_ = 14.9, *p* < 0.05 for intensity and F_(4,16)_ = 54.3, *p* < 0.001 and F_(4,16)_ = 39.6, *p* < 0.05 for ISI. The P2 also showed highly significant linear contrasts for the intensity factor for both the seven regular ISI case (F_(1,4)_ = 114, *p* < 0.001) and five irregular ISI case (F_(1,4)_ = 111, *p* < 0.001). Again no main effect of regularity was observed nor were there any interactions. However, a significant non-linear contrast of ISI by intensity was observed for the seven regular ISI case (F_(1,4)_ = 14, *p* < 0.05) and a linear ISI by intensity contrast for the five irregular ISI case (F_(1,4)_ = 14.4, *p* < 0.05). P2 effects are illustrated in Fig. 4 and Table 2.

### 3.4 Statistical analyses of the source currents

In order to determine source contributions to any observed effects in the measured scalp potentials, current strengths were measured for each of the eight sources at the latencies corresponding to peaks in the GFP associated with the short-latency, i.e. N42 and P52, and long-latency waves, i.e. N1 and P2. For the purpose of a statistical analysis, the source currents were treated as cases in a within-subjects general linear model ANOVA with regularity, intensity and ISI as within-subjects factors (Table 2). In order to obtain a specific measure of what each of the four source zones (i.e. ocular, cerebellar, cingulate and temporal lobe) contributed we also include a between-subjects factor of “zone”, which for the late waves N1 and P2 was restricted to the three cephalic (non-ocular) areas (see Table 3). As noted above, all analyses were conducted with non-transformed data.

**Fig. 5:**
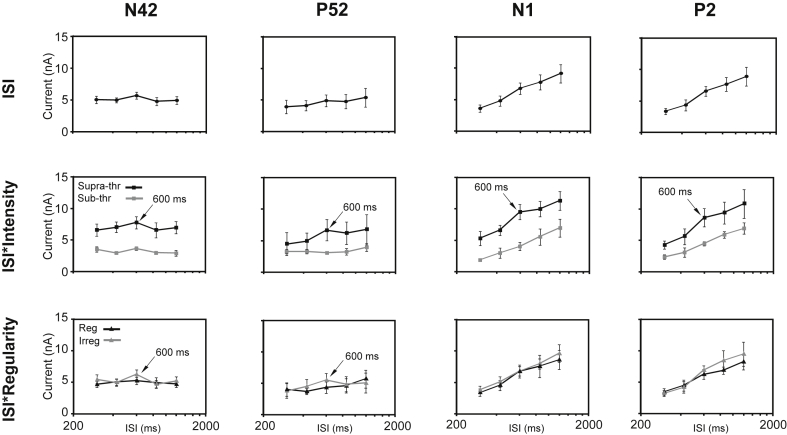
Estimated marginal means (and SEs) for “non-zone effects” from the ANOVAs of source currents for five irregular inter-stimulus intervals (ISIs) measured at latencies of the N42, the P52, the N1 and the P2. (Top) Main effect of ISI, (middle) effect of intensity by ISI and (bottom) effect of regularity by ISI.

**Fig. 6:**
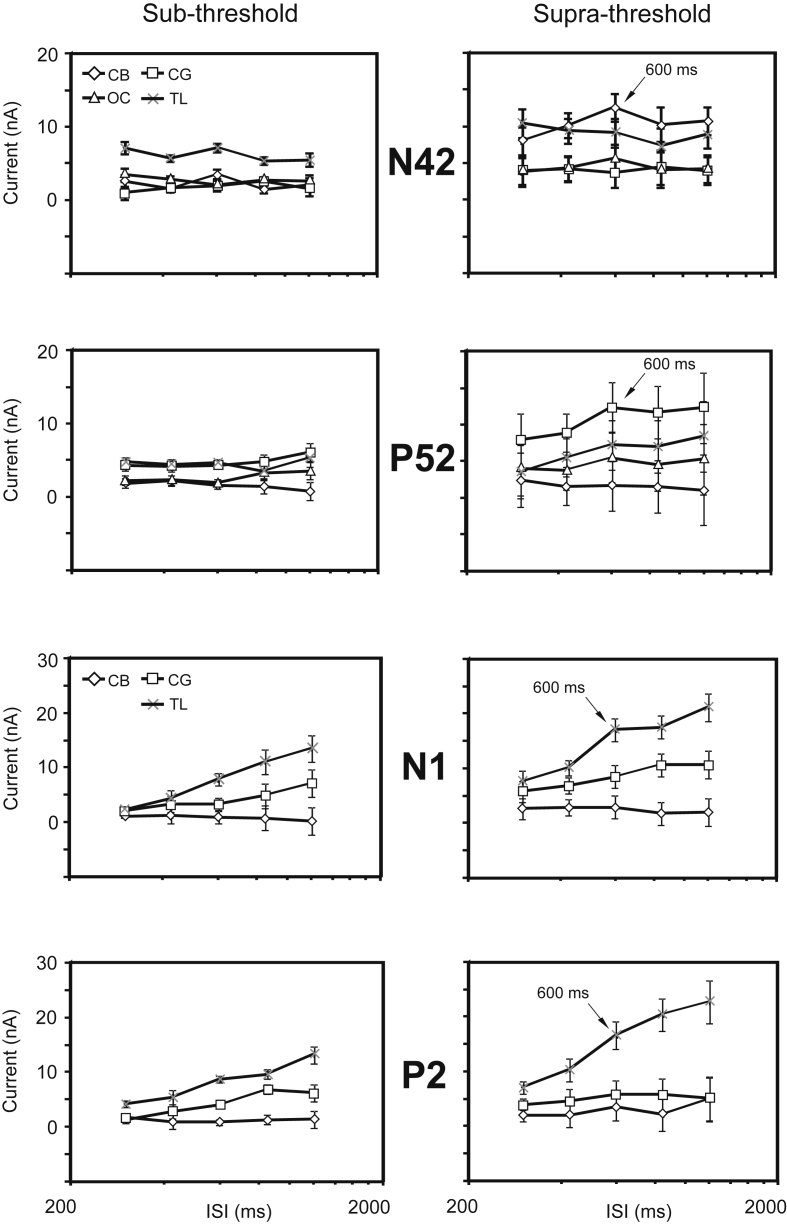
Estimated marginal means (and SEs) for “zone effects” from ANOVAs of source currents for five irregular inter-stimulus intervals (ISIs) measured at latencies of (a) the N42, (b) the P52, (c) the N1 and (d) the P2. (Left) sub-threshold, (right) supra-threshold. The source zones are ocular (OC), cerebellar (CB), cingulate (CG) and temporal (TL).

For the N42 five irregular ISI case the ANOVA yielded main effects of intensity only (F_(1,4)_ = 21.7, *p* < 0.05), consistent with the scalp voltage analysis (Fig. 5). There was in addition an intensity by regularity interaction, (F_(1,4)_ = 10.7, *p* < 0.05), and also an interaction with the intensity factor in the within-subjects contrasts, i.e. a quadratic intensity by regularity by ISI interaction (F_(4,16)_ = 10.0, *p* < 0.05), as well as a linear regularity by ISI by zone contrast (F_(1,4)_ = 7.1, *p* < 0.05) (Table 2, Table 3). The intensity by regularity interactions indicate an overall preference, in the form of larger currents for middle ISIs (i.e. with an ISI of 600 ms) when the stimulation is irregular, but this pattern varies considerably between the zones. At the high intensity the largest contributions to the N42 come from the cerebellum followed by the temporal lobe sources, but with the cerebellar sources showing a clear preference for 600 ms (Fig. 6). The ocular sources also show the 600 ms preference. At the low intensity the cerebellar source drops in magnitude compared to the temporal lobe source, but nevertheless retains a preference for 600 ms. When the ANOVA was applied to the seven regular ISI case (without the regularity factor) the pattern of significance in intensity and ISI was similar, but in addition a significant 4th order intensity by ISI contrast was present (F_(1,4)_ = 21.6, *p* < 0.05).

For the P52 the same analysis as for the N42 for the five irregular ISI case yielded no main effects, even for intensity, but again there were a number of interactions with the intensity factor, in this case intensity by ISI (F_(4,16)_ = 4.9, *p* < 0.05), intensity by regularity, (F_(1,4)_ = 22.6, *p* < 0.01), and intensity by regularity by zone (F_(1,4)_ = 6.3, *p* < 0.05) (Fig. 6). These interactions also show up in the within-subjects contrasts (Table 2, Table 3). Similarly to the N42 the intensity interactions can be interpreted as indicating that although there was no main effect of ISI there was a preference for intermediate ISIs, i.e. at an ISI of 600 ms, depending on regularity and zone, but the patterns of interdependence are distinct (Fig. 5). As for the N42 it would appear that the 600 ms preference is enhanced for irregular stimulation, particularly at the high intensity, however each of the source zones behave differently. In contrast to the N42, for the P52 the cerebellar sources contribute relatively less but the cingulate sources more (Fig. 6), followed by the temporal lobe and ocular sources. At the high intensity the cingulate, temporal lobe and ocular sources all show a 600 ms preference, and this is enhanced by irregularity. The 600 ms preference is absent in all sources at the low intensity. Again the seven regular ISI case replicated the pattern of significance in intensity and ISI.

Turning to the source analysis for the long latency waves, an ANOVA of N1 for the five irregular ISI case with the zone factor restricted to the three non-ocular cephalic sources, yielded main effects of ISI (F_(4,12)_ = 17.8, *p* < 0.05), as well as intensity (F_(1,3)_ = 82.7, *p* < 0.005), and interactions of intensity by zone (F_(2,3)_ = 10.6, *p* < 0.05), and ISI by zone (F_(8,12)_ = 9.1, *p* < 0.05). After removing the linear trend with ISI a preference for 600 ms was once again observed overall for the high intensity but each of the three zones show distinct behaviour as a function of ISI and intensity (Fig. 5). In all cases the temporal lobe sources contribute the largest currents to the generation of the N1 and the 600 ms ISI preference is primarily due to these sources for this wave (Fig. 6). The cingulate sources exhibit an almost pure ISI effect without any sign of the 600 ms preference and the cerebellar source shows the opposite trend of reducing with ISI, but with some evidence of a 600 ms preference at the higher intensity. Unlike the N42/P52 waves regularity has only a small and insignificant effect.

Finally, for the P2 main effects were again obtained for ISI (F_(4,12)_ = 21.5, *p* < 0.005) and intensity (F_(1,3)_ = 11.6, *p* < 0.05), with an interaction of ISI by zone (F_(8,12)_ = 8.4, *p* < 0.05). The ANOVA also yielded a significant between-subjects effect of zone (F_(2,3)_ = 10.3, *p* < 0.05). Overall the P2 analysis gave a similar outcome to the P1. If the linear ISI trend is removed evidence of a preference for 600 ms can be observed overall at the high intensity (Fig. 5), and again the three zones show contrasting behaviours as a function of ISI and intensity (Fig. 6). As for the N1 the largest contributor to the P2 is from the temporal lobe sources, but unlike the N1 both cingulate and cerebellar sources also contribute to the 600 ms preference at the high intensity.

### 3.5 Summary of results

Taking stock of the overall pattern of results (summarised in Table 2, Table 3), within the “non zone” effects, a clear overall pattern of significance is apparent. For the intensity factor all four waves show some effect. The N42 and N1 show intensity effects in both scalp potential and source current analyses although for the P52 and P2 primarily in the scalp potential analyses. For the ISI factor only the long-latency waves show an effect. In most cases for both potential and current analyses the effects are highly significant, and further, for most cases contrasts are also significantly linear. Although we did not use a log-transform on the measurements, these linear contrasts are consistent with the long-latency potentials obeying an ISI power law as the independent variable was linearly spaced on a log-scale. In addition to main effects of intensity and ISI there is also evidence in all four waves of a non-linear contrast interaction between ISI and intensity. Unlike the above, the regularity factor and its interactions yielded little evidence of producing any significant effects, apart from an interaction with intensity in the source current analyses for the N42 and P52 waves and for the interaction with ISI by intensity for the N42. Considering zone effects, a significant main effect of zone appeared only for the P2, consistent with the P2 being dominated by large STG sources. The zone factor also interacted with intensity for the N1 and with ISI for both N1 and P2, again consistent with the STG sources becoming dominant at slow rates (i.e. for longer ISIs) and for the N1 especially at the higher intensity. The evidence of an interaction of the regularity factor for the short latency waves is supported by the zone by regularity by intensity interactions and zone by regularity by ISI contrasts consistent with the sub-cortical sources playing a more complex role for these waves.

## 4 Discussion

### 4.1 Comparison of the present results with the prior literature

The results from analysis of the GFP yielded main effects which are consistent with prior literature (e.g. Picton et al., 1974, Hari et al., 1982, Sams et al., 1993, Carver et al., 2002, Snyder and Large, 2005). These are that for the short-latency waves, which overlap in epoch with the auditory mid-latency waves, the main effect of ISI is non-significant, in contrast to the long-latency waves, which overlap with the late AEPs and their magnetic equivalents, and which show very clear main effects of ISI. This overall pattern of results is apparent at both intensity levels. The apparent power-law observed for the N1 and P2 waves is consistent with prior suggestions of an exponential relationship (Lü et al., 1992, Sams et al., 1993), although a linear ISI relationship has also been indicated (Carver et al., 2002). These main effects were also consistently present in the source current analyses of the same waves where the dominant sources were located in bilateral superior temporal cortex, consistent with our present understanding of the auditory cortical origin of the long latency AEPs (Näätänen and Picton, 1987, Scherg et al., 1989). Our failure to observe main effects of regularity is also consistent with the results of Snyder and Large (2005).

In addition to the above, which replicate prior findings, our data also reveal some novelty in showing, first of all, evidence of non-linear interactions between intensity and ISI in the GFP associated with the long-latency waves, and secondly, evidence from the BESA that vestibular-dependent source generators contribute to the non-linear intensity/ISI interactions in both short and long-latency waves. Some evidence of non-linear effects in short-latency waves has been previously suggested (Carver et al., 2002, Snyder and Large, 2005), especially for the P1, where a specific non-linear ISI effect for intervals around 500 ms may have been due to overlap of adjacent responses (Carver et al., 2002). However, the role of intensity and vestibular dependence was not considered.

### 4.2 Source analyses in comparison to the vestibular imaging literature

The use of three or four pairs of sources to model both short and long-latency effects has been our standard approach in a number of studies over the last decade. In Todd et al. (2008) we were able to provide an account of short-latency VsEPs for both air- and bone-conducted sound where the dominant sources were those associated with the vestibular-ocular reflex (VOR) pathways underlying the generation of the ocular VEMP, but did include frontal generators, including anterior insula. Consistently in these analyses it was found necessary to include concurrent deep sources located close to or in the cerebellum or vestibular brainstem. The vestibular brainstem/cerebellar complex is well-established as a central hub in the pathways controlling the gain of the VOR, including the otolith-ocular reflexes (Büttner-Ennever, 1999). The presence of cerebellar sources in a model which extends to short and long-latency VsEPs is, therefore, entirely plausible. In Todd et al. (2014a) and especially in Todd et al. (2014b) both ocular and cerebellar sources were consistently and independently localized along with additional cortical sources, particularly bilateral regional sources in auditory cortex and mid-line sources most frequently located to the cingulate areas. Numerous imaging studies have demonstrated the existence of a significant vestibular cingulate region (e.g. for review see Lopez and Blanke, 2011, Lopez et al., 2012), and so again is quite plausible as a vestibular-dependent area which may be recruited above the VEMP threshold. In Todd et al. (2014b) the cingulate sources, and in particular a right-hemisphere cingulate source was found to contribute to the N42/P52 wave which we identified as being of vestibular and not cochlear origin. When this modeling approach was applied to the present independent data, once again the same sources, i.e. ocular, cerebellar, cingulate and superior temporal, were obtained in locations which lie close to those obtained in the previous studies. For these reasons we believe that the BESA model we have presented above is robust, consistent and meaningful from a vestibular perspective.

The identified sources are different from some of those suggested by McNerney et al. (2011) who located primary visual, precuneus and pre-motor, as well as temporal lobe sources. One important reason for the differences may have been the latency range of their analysis, which focused on time points of 15 and 20 ms. For example, our earlier study of short-latency VsEPs in Todd et al. (2008) also implicated pre-motor sources. Another may have been the fact that they employed a non-parametric approach in the form of low-resolution electromagnetic tomography (LORETA). Non-parametric approaches are preferred by many practitioners as they require fewer assumptions. However, for LORETA the estimated current density solutions are constrained to cortical grey matter. From a vestibular perspective this limitation is critical as sub-cortical and extra-cephalic sources, e.g. from the musculature around the head and eyes, are likely involved. Thus our cerebellar sources, which are especially active in the early part of the epoch, could be interpreted as being occipital in origin with LORETA.

Recent studies making use of supra-vestibular threshold acoustic stimulation provides independent support for our approach. Using fMRI, Indovina et al. (2015) localised bilateral STG sources which they identified with the parietal insula-vestibular cortex (PIVC). Earlier imaging studies using sound stimulation also indicated this area (Miyamoto et al., 2007, Schlindwein et al., 2008). Given the proximity of our sources to the insula, as well as to auditory cortex, it is likely these correspond to a PIVC zone. Indovina et al. (2015) also localised significant mesial-temporal, i.e. hippocampus, activity which could plausibly also be subsumed in our temporal lobe sources, as well as cerebellar and anterior cingulate sources. The present model did not resolve anterior insula, which was additionally indicated by Indovina et al. (2015), although, as noted above, our earlier analyses of short-latency VsEPs (Todd et al., 2008) did indeed suggest these areas. It is possible that our focus here on the longer-latency potentials and limitations of resolution did not allow us to resolve this region in the present study, its activity likely again being subsumed into the early activity of the large temporal sources.

Some further support for components of our model comes from a BESA model proposed by Kammermeier et al. (2015). Their model, like ours, included pairs of regional sources and an anterior midline source. One pair of sources, which they labelled as parietal operculum, and identified with PIVC, as with Indovina et al. (2015), likely also corresponds to our temporal sources. Another pair which they identified with anterior insula was absent from our present model, as noted above. An important difference with our procedure, however, is that we included both infra-ocular and inion electrodes in the analysis which allowed us to localise ocular and cerebellar sources. It is possible that their frontal sources could include some ocular influence, and indeed experiments with these leads turned off relocalise our ocular sources frontally. Conversely our ocular sources could have absorbed some frontal activity. Although the initial two waves of the OVEMP are completed by 20 ms the ocular sources continue to have some influence potentially due to both later components of the OVEMP and also subsequent EOG induced from vestibular evoked eye movements. We believe that infra-orbital electrodes are essential for source analysis under conditions where OVEMPs can be expected to occur, even if resulting ocular sources may mask some frontal activity.

### 4.3 A vestibular locomotor hypothesis to explain the non-linear interactions

When we applied our present model to each of the conditions we could see distinct and definite patterns for each of the generators for each of the four waves. For the N42 at the supra-threshold intensity the cerebellar sources dominated, showing a distinct preference for 600 ms and dropping off for longer ISIs. For the P52 the cingulate source was the largest contributor, again showing a distinct 600 ms ISI advantage. Curiously for both the N42 and P52 waves the dominant sources show a higher peak at 600 ms in the irregular condition, a point we return to later. For both the N1 and P2 waves the superior temporal lobe/PIVC sources were the dominant generators, also exhibiting evidence for around a 600 ms preference, especially if the linear ISI trend is removed. All four cases independently show evidence that one or more of the vestibular-dependent generators have a preference for the middle ISIs, usually 600 ms, and this provides the explanation for the observed non-linear ISI intensity interactions. This pattern of results may also explain why the non-linear interaction was not observed in the scalp voltage analysis for the N42 and P52 cases. The GFP is computed from the all 70 scalp electrodes which will inevitably be dominated by the cortical sources which are closest to the surface. Thus for the N42/P52 where the main origin of the interaction is sub-cortical or deeper within the brain, the interaction will be less significant given their relative remoteness compared to the bilateral temporal lobe sources.

One possible explanation for the presence of the apparent preference for middle ISIs in the vestibular-dependent sources is that the vestibular system is highly tuned to stimulus rates which coincide with those frequencies typically found during locomotion. The cadence of locomotion has been studied extensively. For example, Whittle's (1991) classic work on gait analysis gives a range of cadence for walking to be approximately inter-step intervals of 440–660 ms, or step rates of about 1.5–2.3 Hz. Many studies have investigated the relationship between locomotion and other human natural frequencies including spontaneous and preferred tempo (e.g. Harrison, 1941, Mishima, 1965; for review see Fraisse, 1982). More recently, MacDougall and Moore (2005) in an accelerometry analysis of a number of locomotor and non-locomotor activities confirmed a well-defined, sharp distribution of locomotor accelerations of around 2 Hz (range 1.7–2.2 Hz). During locomotion, in particular the vertical head acceleration follows the step frequency of the body and, therefore, reflects the 2 Hz frequency of cadence. In contrast the magnitude of lateral (i.e. inter-aural) and naso-occipital accelerations are significantly lower, and indeed the lateral acceleration reflects the lower frequency of whole body sway associated with half the step frequency (i.e. about 1 Hz). Of particular relevance to the present study, the vertical head accelerations associated with locomotion will be sensed by the saccules which have their hair-cells morphological polarised in the dorsoventral axis (Benson, 1982). Consistent with the vestibular tuning hypothesis it has been demonstrated that saccular irregular spontaneous afferent units have a high-pass gain with a maximum at 2 Hz in the range measured (Fernández and Goldberg, 1976). This is reflected in the frequency gain of the otolith-spinal, -collic and -ocular reflexes (Hirasaki et al., 1999, Moore et al., 2001), which are put under particularly high demand during locomotion.

In our study we employed air-conducted sound stimuli with a frequency distribution at around 500 Hz which has been shown to optimally activate both species of VEMP (Todd et al., 2000, Todd et al., 2009). While it is generally agreed that the acoustically responsive receptors are otolith irregular afferents (McCue and Guinan, 1994), there is less agreement on whether they are primarily saccular or utricular in origin (e.g. Todd, 2014). It has been suggested that the OVEMP is predominantly utricular, while the cervical VEMP saccular, but the contribution to central vestibular generators is unknown and almost certainly there will be a mix of both otolith receptor species depending on individual thresholds (Govender et al., 2015). There is evidence that for AC sound, as opposed to BC sound, the utricular threshold is some 20 dB above that of the saccule (Young et al., 1977). It is likely therefore that our supra-threshold stimuli recruited a significant proportion of saccular irregular afferents of the type which have been shown to have a higher gain at stimulation rates of about 2 Hz.

The present results, and the vestibular locomotor hypothesis which we offer to explain them, are also relevant to the field of rhythm perception. The overlap in the distribution of beat rates in music and step rates in locomotion is well known (Fraisse, 1982, van Noorden and Moelants, 1999), a point also raised by MacDougall and Moore (2005). Such observations have led to the proposal of a “sensory-motor” theory of rhythm perception in which it was hypothesised that the motor and vestibular systems play a central role in mediating the perception of an auditory rhythm, even in the absence of explicit movement (Todd, 1999, Todd et al., 2002, Todd and Lee, 2015a). The vestibular/rhythm connection may also offer an explanation for the increased response in the irregular condition at 600 ms for the N42/P52 cases, as noted above, since it has been shown that repetitive isochronous rhythms show a reduction in electrophysiological response magnitude compared to more complex or irregular rhythms (Todd and Lee, 2015b).

The sensory-motor theory has received considerable supportive evidence in the last decade from imaging studies which have shown motor planning areas, including the supplementary and cingulate motor areas, activated during the passive perception of beat based rhythms (Chen et al., 2008, Grahn and Brett, 2007, Zatorre et al., 2007). These areas overlap in location with our cingulate sources. Evidence to support the theory also comes from behavioural studies showing that the vestibular system contributes to auditory rhythm perception (Phillips-Silver and Trainor, 2008, Trainor et al., 2009). We also note that most rhythms which have a beat are associated with dancing, another locomotor-related activity, and dance music is often experienced at intensities which are supra-threshold for vestibular activation (Todd and Cody, 2000). However, further work comparing individual gait and movement patterns with their individual VsEP profiles would more strongly substantiate the vestibular locomotor hypothesis.

## Figures and Tables

**Table 1:** TTCs and possible origin for BESA model.

Location			Zone	L-orientation			R-orientation			Possible origin
X	Y	Z		X	Y	Z	X	Y	Z	
+-31	63	−27	OC	–	–	–	–	–	–	EOEMs (OVEMPs) + Retina/Cornea (EOG)
+-5	−9	44	CG	0.1	−0.2	1.0	0	1.0	0.3	CG/PCL/MFG BA 24/31/6
+-45	−26	2	TL	–	–	–	–	–	–	STG/MTG/Insula BA 22/41/13/21
+-11	−78	−17	CB	−0.1	−1.0	−0.3	−0.3	0.2	0.9	Posterior Lobe/Declive

Abbreviations: BA Brodman area, CB cerebellum/cerebellar, CG cingulate gyrus/cingulate, EOEM extra-ocular eye muscles, EOG electro-ocular gram, MFG medial frontal gyrus, MTG medial temporal gyrus, OC ocular, OVEMP ocular vestibular evoked myogenic potential, PCL paracentral lobule, STG superior temporal gyrus, TL temporal lobe.

**Table 2:** Summary of ANOVA results: non zone effects.

Effects	Wave	Scalp voltage		Source current		Comments
		(7)	(5)	(7)	(5)	
Intensity	N42	*	2 *	*	*	All waves show some effect of stimulus intensity
	P52	*	3 *	ns	ns	
	N1	*	*	3 *	3 *	
	P2	*	3 *	ns	*	

ISI	N42	ns	ns, 1^st^*	ns	ns	Linear (1^st^ order) contrasts imply a power law ISI effect for N1/P2
	P52	ns	ns	ns	ns	
	N1	3 *, 1^st^ 2 *	3 *, 1^st^ 3 *	3 *, 1^st^ 3 *	*, 1^st^ *	
	P2	4 *, 1^st^ 4 *	4 *, 1^st^ 4 *	*, 1^st^*	3 *	

Intensity ^x^ ISI	N42	ns	ns	ns, 4^th^ 2 *	ns	All waves show evidence for a non-monotonicity/ISI preference at around 600 ms, especially for high intensity.
	P52	ns	ns	ns, 3^rd^ *	*	
	N1	ns, 5^th^ *	ns, 3^rd^ 3 *	ns	ns	
	P2	ns, 4^th^ *	ns, 1^st^*	ns, 3^rd^ 2 *	ns	

Regularity	N42		ns		ns	
	P52		ns		ns	
	N1		ns		ns	
	P2		ns		ns	

Regularity ^x^ Intensity	N42		ns		*	N42/P52 show regularity intensity interactions in current sources
	P52		ns		2 *	
	N1		ns		ns	
	P2		ns		ns	

Regularity ^x^ ISI	N42		ns		ns	
	P52		ns		ns	
	N1		ns		ns	
	P2		ns		ns	

Regularity ^x^ Intensity ^x^ ISI	N42		ns		ns, 2^nd^ *	For N42 irregularity sharpens preference for 600 ms at the high intensity
	P52		ns		ns	
	N1		ns		ns	
	P2		ns		ns	

Abbreviations: * p < 0.05, 2* p < 0.01, 3* p < 0.005, 4* p < 0.001, 1^st^ first order (linear), 2^nd^ second order (quadratic), 3^rd^ third order (cubic), 4^th^ fourth order, ns p ≥ 0.05. For cells with two entries the second entry is the contrast. All effects p < 0.01 are highlighted in bold.

**Table 3:** Summary of ANOVA results: Zone effects.

Effects	Wave	Source current		Comments
		(7)	(5)	
Zone	N42	ns	ns	
	P52	ns	ns	
	N1	ns	ns	
	P2	*	*	P2 TL dominant overall

Zone ^x^ Intensity	N42	ns	ns	
	P52	ns	ns	
	N1	ns	*	N1 TL dominant at high intensity
	P2	ns	ns	

Zone ^x^ ISI	N42	ns	ns	
	P52	ns	ns	
	N1	*, 1^st^2 *	*, 1^st^*	N1 TL dominant at long ISIs
	P2	ns	*, 1^st^*	P2 TL dominant at long ISIs

Zone ^x^ Intensity ^x^ ISI	N42	ns	ns	
	P52	ns	ns	
	N1	ns	ns	
	P2	ns	ns	

Zone ^x^ Regularity	N42		ns	
	P52		ns	
	N1		ns	
	P2		ns	

Zone ^x^ Regularity ^x^ Intensity	N42		0.05	N42 regularity/intensity effects mainly due to CB P52 regularity/intensity effects mainly due to CG + OC
	P52		*	
	N1		ns	
	P2		ns	

Zone ^x^ Regularity ^x^ ISI	N42		ns, 1^st^*	N42 effects mainly due to CB
	P52		ns	
	N1		ns	
	P2		ns	

Zone ^x^ Regularity ^x^ Intensity ^x^ ISI	N42		ns, 3^rd^*	N42 effects mainly due to CB
	P52		ns	
	N1		ns, 1^st^*	
	P2		ns	

Abbreviations: * p < 0.05, 2* p < 0.01, 3* p < 0.005, 4* p < 0.001, 1^st^ first order (linear), 2^nd^ second order (quadratic), 3^rd^ third order (cubic), 4^th^ fourth order, ns p ≥ 0.05. Abbreviations in comments as for Table 1. For cells with two entries the second is the contrast. All effects p < 0.01 are highlighted in bold.
